# Genomic-based genetic parameters and genome-wide association studies for productive and reproductive traits in Beef-on-Dairy crossbreds

**DOI:** 10.3389/fgene.2025.1530310

**Published:** 2025-06-05

**Authors:** R. H. Ahmed, C. Schmidtmann, J. Mugambe, G. Thaller

**Affiliations:** ^1^ Institute of Animal Breeding and Husbandry, Christian-Albrechts-University Kiel, Kiel, Germany; ^2^ IT Solutions for Animal Production (vit), Verden (Aller), Germany

**Keywords:** beef-on-dairy, haplotypes, birth weight, calving difficulty, gestation length

## Abstract

**Background:**

Beef on Dairy (BoD) calves are born from the crossing of dairy cows with beef breeds. The genetic architecture of these calves differs significantly from the parent breeds due to heterosis and other dominance effects. Identification of the genomic regions associated with traits in BoD calves and the inheritance pattern of these regions can assist in the selection process. We conducted a genome-wide association study (GWAS) for Belgian blue and Angus crossbreds born from a Holstein dam, incorporating additive and dominance effects to identify genomic regions associated with birth weight, calving difficulty, and gestation length. Additionally, a haplotype-based GWAS was performed to compare the effectiveness of these two different methodologies and to identify the parental origin of the haplotypes based on similar allelic patterns between crossbred and parental breeds.

**Results:**

The heritability estimates for birth weight, calving difficulty, and gestation length were 0.29 (±0.03), 0.36 (±0.04), and 0.09 (±0.03), respectively. Using SNP-based GWAS for birth weight, a genomic region containing the *GABRG1* gene on BTA 6 was identified. In addition, the haplotype-based analysis identified three more genes (*CSER1*, *FAM13A*, and *LCORL*) associated with birth weight. Incorporating dominance effects into the GWAS model led to the identification of an additional gene, *SPP1*, related to birth weight. For calving difficulty, SNP-based GWAS in Angus crossbreds revealed a genomic region containing the *KCNIP4* gene. Most of the haplotypes associated with these traits originated from the three parental breeds, but six unique haplotypes for Angus and Belgian blue were identified.

**Conclusion:**

Based on this study, Haplotype GWAS was found to have superior statistical power in the identification of associated genomic regions in BoD crossbreds. However, for traits such as calving difficulty, SNP-based GWAS proved to be more effective. Both approaches are essential for the identification of genomic regions associated with traits of interest in BoD calves.

## Introduction

Mating dairy cows with beef bulls (Beef-on-Dairy, BoD) has become increasingly popular in recent years. This trend is aimed at producing calves with higher monetary value because such calves are expected to have better growth rates and superior carcass characteristics compared with purebred dairy calves (Bittante et al., 2023). However, at the same time, risk of calving difficulty is higher when applying BoD. The birth weight (BW) of the calf and the gestation length (GL) of the dam are well-known factors that influence calving difficulty (CD) (Kargo et al., 2014; Jenkins et al., 2016). In this regard, selection of beef sires to be used in dairy herds, which show a balanced genetic potential for optimal growth and calving ease are of high interest for farmers. To identify such bulls in view of negative genetic correlations between birth weight and calving ease requires a better understanding of the genetic architecture of relevant traits. This is especially important in crossbreeding systems where the performance of crossbred calves may vary from the purebred counterparts also due to heterosis and breed complementary effect (González-Diéguez et al., 2020; Khansefid et al., 2020). In the last 2 decades, genome-wide association studies (GWAS) have demonstrated their large potential to identify genes associated with different traits in cattle breeding, thereby pinpointing the selection process (Zhang et al., 2022). Traditionally, GWAS in purebred populations focuses on the additive effects associated with single-nucleotide polymorphisms (SNP), but for crossbred populations with differences in genetic architecture, the inclusion of dominance effects can help in identifying quantitative trait loci (QTL) influencing traits with low to moderate heritability (*h* ^2^) (Zhang et al., 2008). Dominance described as non-additive interactions of different alleles at a specific locus is a major phenomenon to explain heterosis effect in animal breeding (Visscher et al., 2000). Especially in crosses of two different breeds interactions of differently selected alleles can account for a major fraction of the genetic variation (Cui et al., 2023). Historically, estimation of dominance based on pedigree data has been notoriously difficult due to the requirement of large numbers of full-sib families. But since the development and accessibility of large SNP panels, reliable estimation of dominance effects has become possible (Vitezica et al., 2013). Along with the SNP-based GWAS, GWAS based on haplotypes can also be of significant interest when analyzing crossbred populations due to the inheritance of such blocks with lower probability of recombination (Gabriel et al., 2002; Bovo et al., 2021). It has been shown that GWAS based on haplotypes can outperform SNP-based GWAS by exploiting aggregated effects of consecutive SNPs for quantitative traits, where individual loci usually have a small effect (Bickel et al., 2011). Additionally, haplotype blocks can be used to determine the parental origin of the regions with significant association for the traits of interest (Vandenplas et al., 2016). This can assist in making informed decisions about the selection of the sires with desired genetic architecture for the traits of economic interests. This study compares different approaches of GWAS based on SNP and haplotype blocks in BoD crossbreds for the traits BW, GL and CD and identifies genomic regions harbouring relevant genes associated with the traits. GWAS analyses are based on single SNPs as well as on haplotypes. In addition, haplotype blocks with significant association are traced to the respective parent breed to identify the origin of the gene variants in the crossbred population.

## Materials and methods

### Data and trait description

This study used 4,118 BoD crossbred calves sired by Belgian Blue (WBB) or Angus (ANG) and born to Holstein (HOL) dams. Between December 2021 to December 2023, the weight of calves was recorded once at the age of 0–40 days on 225 dairy farms in Schleswig-Holstein, Germany. Information regarding the insemination date and parity number of the dam, birth date and sex of the calf, type of birth (singleton or twin) and calving difficulty (CD) recorded on the official German scale (Arbeitsgemeinschaft Deutscher Rinderzüchter; organization of cattle production in Germany) and converted into binary scale (coded, 0 = no difficulty, 1 = difficult calving) was available. Gestation length (GL) was calculated as the duration (in days) between the date of insemination and the date of calving. Recorded values for weight and gestation length deviating ±4 SD from the mean were removed to filter for outliers. Additionally, twin calvings and farms with less than two observations were excluded from the analysis. After editing, 285 animals were excluded from the analysis and the final dataset contained 3,833 calves from 116 farms.

3,530 crossbred calves were genotyped using EuroG MD BeadChip (Illumina Inc). Quality control of genotypes was performed separately within ANG and WBB crossbreds, respectively, using PLINK 1.9 (Chang et al., 2015). SNPs with a minor allele frequency of <1%, a call rate lower than 90% and SNPs that deviate from the Hardy–Weinberg equilibrium at threshold of 1 × 10^−6^ along with variants located on sex chromosomes were excluded from the analysis (Supplementary Table S1).

### Estimation of birth weight of calves

In order to approximate the birth weight (BW) of the calves recorded not at the day of birth, a correction of measured weight was performed within each breed using the following linear regression model (Equation 1):

$$y_{g}=\mu +{\mathrm{AGE}}_{g}+e_{g}$$ (1)

where y_g_ was the measured weight (kg) of a calf within the sire breed WBB or ANG, AGE_g_ represented the regression of calf’s age at the day of measurement (g = 0, … ,40) and e_.g.,_ was the residual term. The model was fitted in R (Becker et al., 1988) using the package lme4 (Bates et al., 2015). BW of the calf was calculated by subtracting the average predicted weight gain over the period of time from the measured weight of the calf.

### Analysis of population structure

Population stratification of the animals under study was examined using principal component analysis (PCA) in the individual breeds but also in the combined population (COM) via PLINK 1.9 (Chang et al., 2015). The first three components of PCA were used for visualization.

### Haplotype phasing and block construction

Haplotype phasing was conducted using SHAPEIT v2 (Delaneau et al., 2013) for each autosomal chromosome with default parameters for MCMC iteration combined with pedigree information using–duoHMM option to apply pedigree based *post hoc* haplotypes correction (O’connell et al., 2014). Haplotypes for *Bos taurus* autosomes (BTA) 1 to 29 were combined and converted into the binary format using the R package GHap (Utsunomiya et al., 2016). For further analysis, haplotype blocks were generated with a sliding window of five consecutive SNPs as this approach has been found to have the highest power to detect associated QTLs (Braz et al., 2019).

### Variance components and statistical model for GWAS

Genetic parameters for BW, GL and CD were estimated by using both univariate additive models and dominance models. SNP-based variance components were estimated using restricted estimation of maximum likelihood (REML) using the software GCTA (Yang et al., 2011) while narrow sense heritability for the trait was estimated as $h^{2}=\sigma_{a}^{2}/\left(\sigma_{p}^{2}\right),$ where $\sigma_{a}^{2}$ represents the additive genetic variance and $\sigma_{p}^{2}$ is the phenotypic variance.

GWAS were performed for each trait using univariate single SNP regression mixed linear models in GCTA (Yang et al., 2011). The additive model was (Equation 2):

y=Xb+Wa+Zu+e (2)

where **y** is the vector of phenotypes (BW, GL or CD); **b** is a vector of fixed effects (herd-year-season, sex, parity number, first three principal components as covariates), **a** is the vector of polygenic effects with $a∼N\left(0,G\sigma_{a}^{2}\right)$ , where **G** is the genomic relationship matrix (grm) (Yang et al., 2010) and $\sigma_{a}^{2}$ is the additive genetic variance, **u** is the vector for SNPs for homozygous major and minor (0,2) and heterozygous alleles (1) and **e** is the random residual effect with $e∼N\left(0,I\sigma_{e}^{2}\right)$ , where $\sigma_{e}^{2}$ is the residual variance and **I, X**, **W** and **Z** represent incidence matrices for **b**, **a, u** and **e** respectively. For the GWAS model based on haplotypes, **a** represents the vector of polygenic effects, where the estimation of G is based on haplotypes.

For the dominance model (Equation 3), a modified version of Equation 2 was used:

y=Xb+Wa+Wd+Zu+e (3)

where **d** is the vector of dominance effects with $d∼N\left(0,D\sigma_{d}^{2}\right)$ , where **D** is the dominance grm (Equation 4) defined as

$$D_{jk}=\frac{1}{m}\sum_{i}\left(w_{D\left(ij\right)}w_{D\left(ik\right)}\right)$$ (4)

where **m** is the number of SNPs, *w*_*D(ij)*_ and *w*_*D(ik)*_ dominance encoded genotypes, $\sigma_{d}^{2}$ is the dominance variance and **u** is the vector with the modified version of SNP coding (homozygous (0) and heterozygous (1) alleles). All the remaining variables are the same as in the Equation 2. Dominance variation at all SNPs was defined as $\delta^{2}=\sigma_{d}^{2}$ /( ${\sigma_{a}^{2}+\sigma }_{d}^{2}+\sigma_{e}^{2}$ ). The significance threshold for the SNPs was determined using Bonferroni correction, where a p-value of *P* < (0.05/N), with N representing the number of SNPs or pseudo-SNPs analyzed in the GWAS, indicates statistically significant associations. A suggestive association level was set at *P* < (1/N).

### Linkage disequilibrium and annotation of associated SNPs

Linkage disequilibrium (LD) for each crossbred population was measured as the correlation coefficient r^2^ (Hill and Robertson, 1968) by using PLINK v1.9 (Chang et al., 2015). LD decay between adjacent markers up to the distance of 500 kb was visualized in R using package ggplot2 (Wickham, 2011). For the functional annotation of SNPs with significant associations, base pair position was checked within 150 kb up- and downstream of the respective SNPs against the genome assembly *Bos taurus* UMD 3.1.1 to find relevant genes in the region. This distance was defined based on the LD decay (Qanbari, 2019), where the highest drop in first 150 kb of distance was observed in both crossbred populations (Additional file 1: F1). For haplotype blocks, screening for genes was limited to the significantly associated blocks in the analysis. The origin of significant haplotype blocks was determined by comparing them with parental haplotypes. This was achieved by matching the blocks with the corresponding dam and sire haplotypes with same block positions and allelic patterns to confirm their parental origins (Eiríksson et al., 2021a).

## Results

### Descriptive statistics

Descriptive statistics for the crossbred calves are depicted in Table 1. The distribution of the calves per parity number and age group at weight measurement is shown in Additional file 1: F2 & F3. The majority of calves belonged to WBB (Supplementary Table S2). These crossbreds had slightly higher daily weight gain (0.77 kg) compared to ANG crossbreds (0.66 kg). Moreover, calves from WBB had higher birth weight (48.5 kg ± 7.2) and longer gestation length (281.4d ± 4.7) compared to ANG sired calves (BW: 45.2 kg ± 7.2; GL: 280.1d ± 4.7). Incidences of calving difficulty were higher for ANG crossbreds, where 12.5% of calvings were labeled as difficult calving compared to WBB crossbreds with 11.0% difficult calvings.

**TABLE 1:** Descriptive statistics of recorded traits of crossbred calves by sire breed.

Breed	Nr. calves (genotyped)	Gestation length (d)		Parity		Birth weight* (kg)		Calving difficulty
		Mean	SD	Mean	SD	Mean	SD	%
ANG	852 (801)	280.0	4.9	2.2	1.3	45.0	7.5	12.5
WBB	2981 (2729)	281.5	4.8	3.3	1.1	48.4	7.7	11.0
COM	3833 (3530)	281.2	4.8	3.2	1.6	74.7	7.4	11.4

### Principal component analysis

**FIGURE 1:**
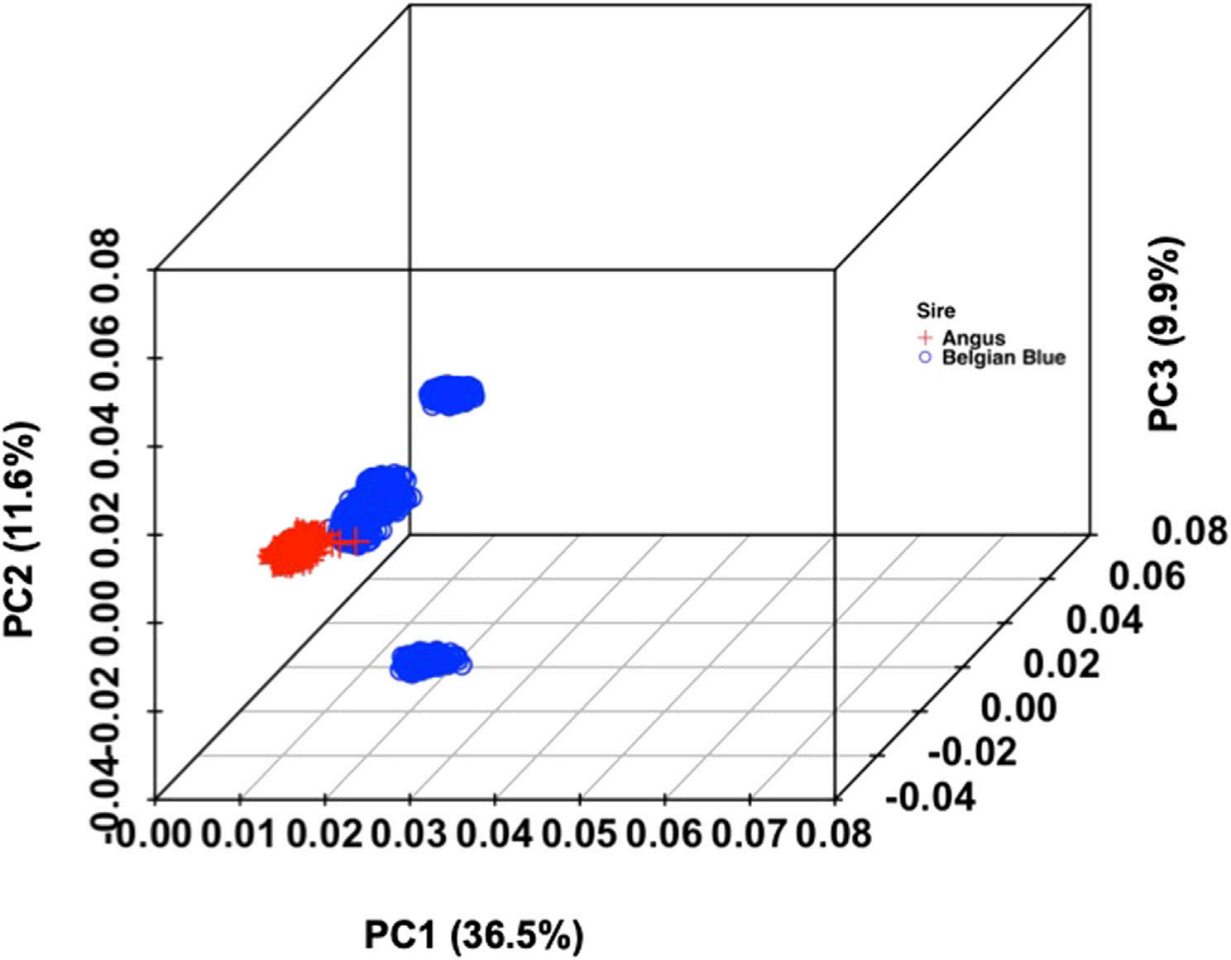
Population structure of the crossbred calves.

Based on the PCA (Figure 1), clear clusters were identified. PC1 and PC2 collectively explained 48% of the variation in the combined data. Slight overlapping was observed for crossbred population based on PC1 and PC2 but with the addition of PC3 (58% variance), distinct clusters were observed across two studied populations (Additional file 1: F3, F4, F5)

### Haplotype blocks and variance components

Descriptive statistics for haplotype blocks are shown in Table 2. In total 11,160 haplotype blocks were created. In the combined population, highest number of haplotype blocks were observed on BTA1 (n = 726) while the lowest number of blocks were found on BTA28 (n = 210). Further details regarding haplotype blocks and number of markers per chromosome are presented in Additional file 1: F6, F7.

**TABLE 2:** Descriptive statistics of haplotype blocks in crossbred population.

	Population		
	COM	ANG	WBB
Number of haplotype blocks	11,160	11,160	11,160
Average blocks per chromosome	384.8	384.8	384.8
Number of pseudo-SNPs before QC	212,770	156,114	195,654
Number of pseudo-SNPs after QC	111,394	98,401	103,933

Estimates of heritability (s.e.) for BW, GL and CD in the combined population using the additive model were 0.29 (0.03), 0.36 (0.04) and 0.09 (0.03), respectively. For WBB, Dominance variation at all SNPs $\left(\delta^{2}\right)$ for BW and GL was close to zero (Table 3), while for CD it was estimated to be 0.08 (0.07) (Table 3; Supplementary Table S3).

**TABLE 3:** Heritability estimates for birth weight, calving ease and gestation length.

Breed	Trait	Additive model	Dominance model	
		*h* ^2^_a_	*h* ^2^_a_	*h* ^2^_d_
COM	BW	0.28 (0.03)	0.27 (0.04)	0 (0.05)
	CD	0.11 (0.03)	0.10 (0.03)	0.08 (0.06)
	GL	0.36 (0.04)	0.36 (0.04)	0.001 (0.05)
ANG	BW	0.36 (0.12)	0.33 (0.13)	0.12 (0.22)
	CD	0.23 (0.13)	0.16 (0.13)	0.25 (0.21)
	GL	0.50 (0.11)	0.46 (0.13)	0 (0.21)
WBB	BW	0.26 (0.04)	0.26 (0.04)	0 (0.07)
	CD	0.07 (0.03)	0.06 (0.04)	0.08 (0.07)
	GL	0.35 (0.04)	0.34 (0.05)	0.01 (0.07)

### Genome-wide associations of traits

#### Birth weight

**FIGURE 2:**
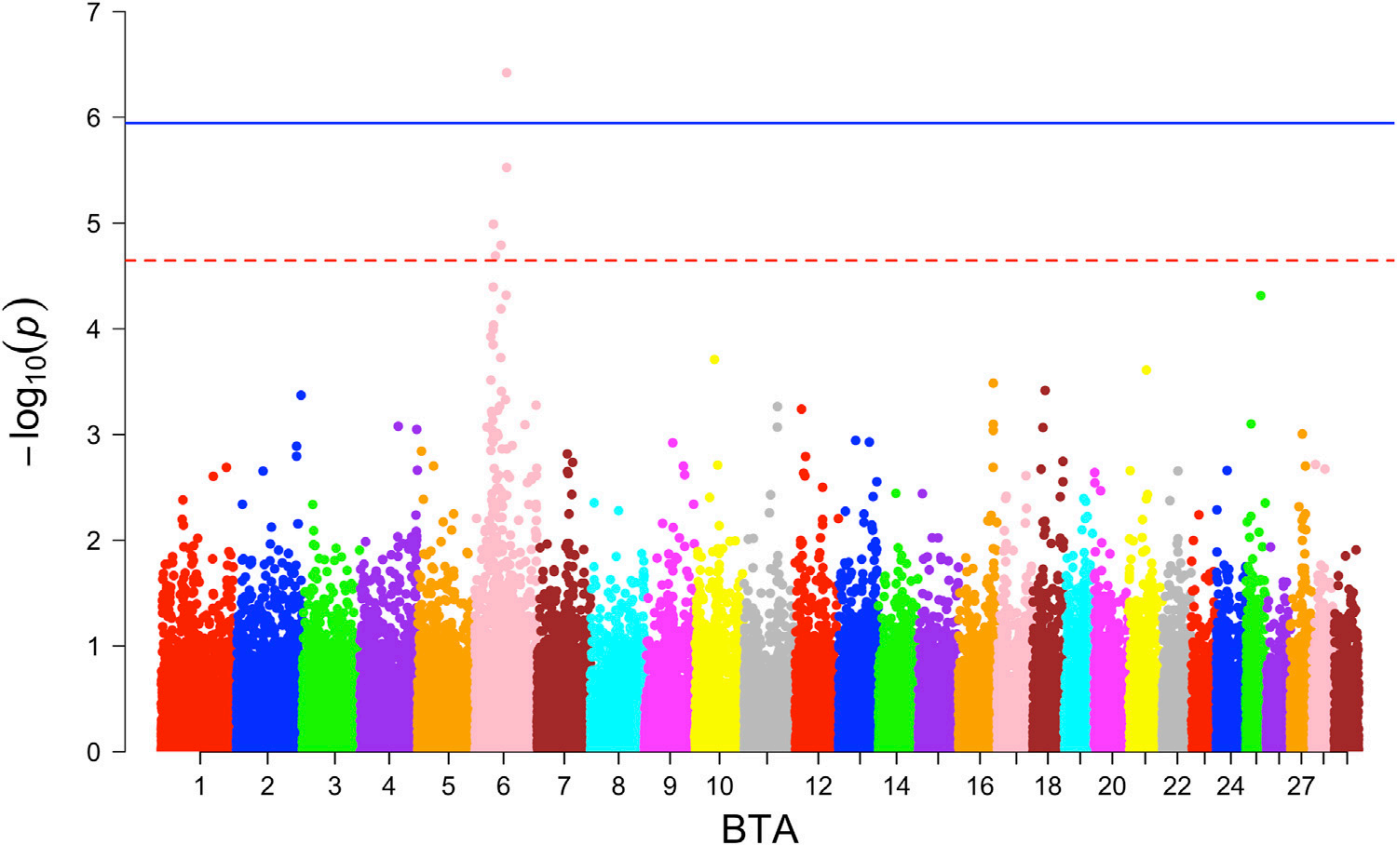
Manhattan plot for combined population GWAS for birth weight.

In the GWAS with the combined crossbred population, one significantly associated SNP on BTA 6 around 6.26 Mb was found based on the additive model (Figure 2). This SNP falls in the region of gene *GABRG1*, which is responsible for the regulation of the ion channel and receptor activity along with the influence on puberty development (Tahir et al., 2021).

At the suggestive significance level, four SNPs were identified through the additive model, while an additional SNP on BTA 6 at 38.13 Mb was identified through the dominance model (Table 4).

**TABLE 4:** Description of SNPs with significant association with traits.

Trait	Model	Breed	CHR	SNP	*P-value*
BW	Additive	COM	6	BTB-01601414	3.78e-07
BW	Additive	COM	6	Hapmap59322-rs29015787	2.99e-06
BW	Dominance	COM	6	Hapmap27072-BTC-033816	3.83e-06
CD	Additive	ANG	6	BTB-00251468	5.23e-06
GL	Additive	WBB	27	Hapmap50420-BTA-62223	1.75e-06
GL	Additive	WBB	27	BTB-01868033	7.93e-06

For the BW, GWAS based on haplotypes revealed eight haplotype blocks on BTA 6, where three additional genes (*CCSER1*, *FAM13A*, *LCORL*) are located (Table 5). Furthermore, 14 haplotype blocks within 10 unique regions were identified (Supplementary Table S4) with suggestive association. Along with the previously mentioned three genes, seven more genes (*SPP1, GABRG1, HERC6, LOC104972722, ADGRL3, SNCA, and PPARGC1A*) were found in the associated genomic regions.

**TABLE 5:** Description of haplotypes with significant association with traits.

Trait	Breed	CHR	Haplotype	*P-value*
BW	COM	6	B119_33128133_33284794	1.78e-06
BW	COM	6	B122_34686041_35147153	1.38e-07
BW	COM	6	B211_56278798_56464060	9.01e-06
BW	COM, ANG	6	B130_37399296_37501365	1.68e-08
BW	COM, WBB	6	B134_38133743_38262298	1.68e-08
BW	COM, ANG	6	B136_38576012_38825835	2.22e-10
BW	COM, ANG	6	B137_38869785_39069719	1.52e-09
BW	COM, ANG, WBB	6	B139_39257620_39438580	3.58e-11
BW	COM, WBB	6	B203_54237782_54390661	6.35e-06
BW	ANG	6	B128_36895328_37066212	4.07e-06
BW	ANG	6	B152_41914851_42084330	8.68e-07
BW	ANG	6	B156_42628140_42782178	9.61e-06
BW	ANG	6	B165_44850990_44991839	7.38e-06
BW	ANG	6	B172_46702267_46867937	7.72e-06
BW	WBB	6	B247_66394378_66509207	3.42e-06
CD	COM	6	B208_55496362_55594668	5.84e-07

#### Calving difficulty

**FIGURE 3:**
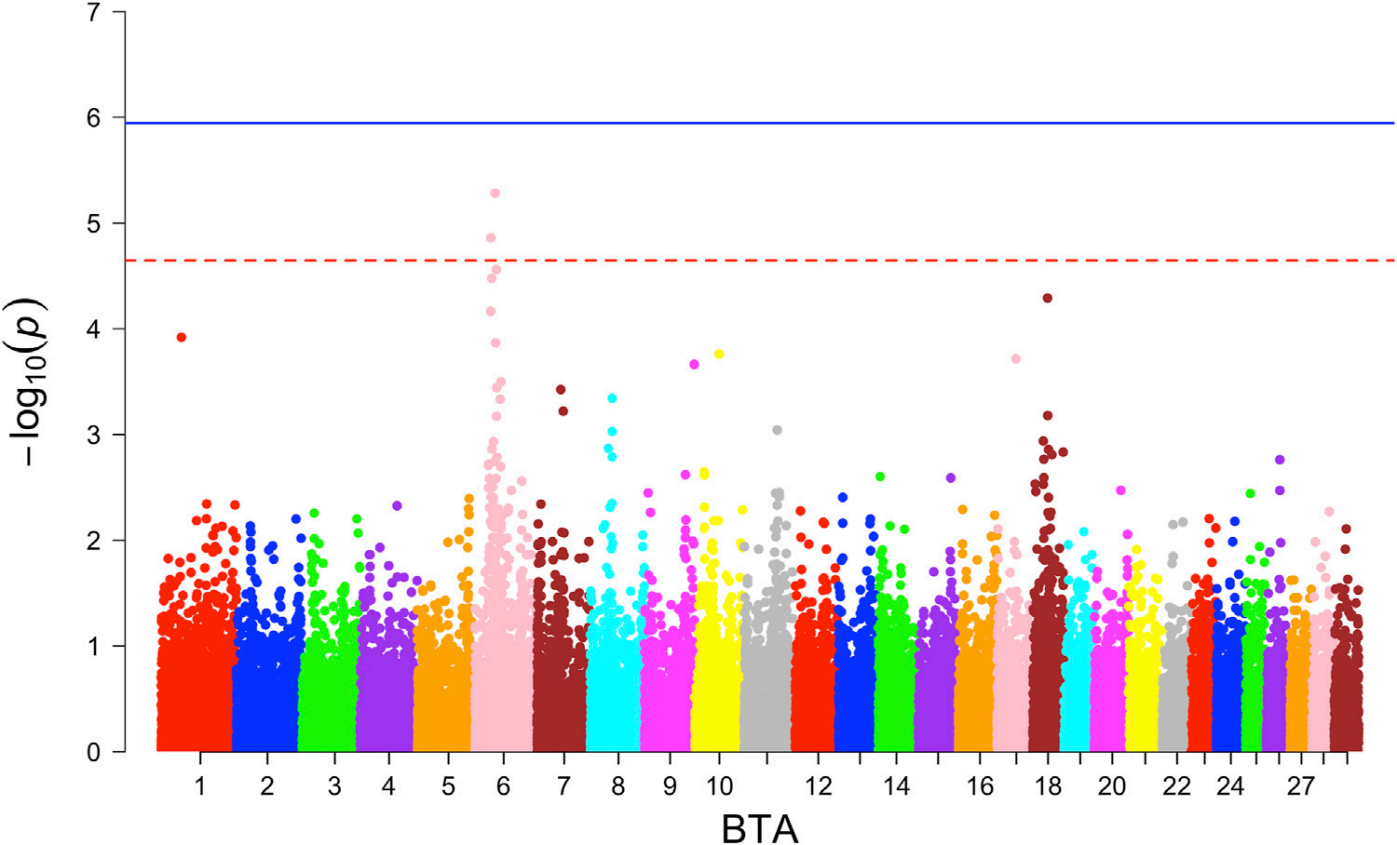
Manhattan plot for Angus crossbred population GWAS for calving difficulty.

Neither additive nor dominance models identified SNPs with significant associations for the combined or individual WBB crossbred population. In the ANG crossbreds, two SNPs were identified on BTA 6 through the additive model (Figure 3) with the suggestive level of association, where SNP at 42.05 Mb harbouring the *KCNIP4* gene in nearby region. Haplotype-based GWAS identified one haplotype block with suggestive association in the combined population (Supplementary Table S4).

#### Gestation length

**FIGURE 4:**
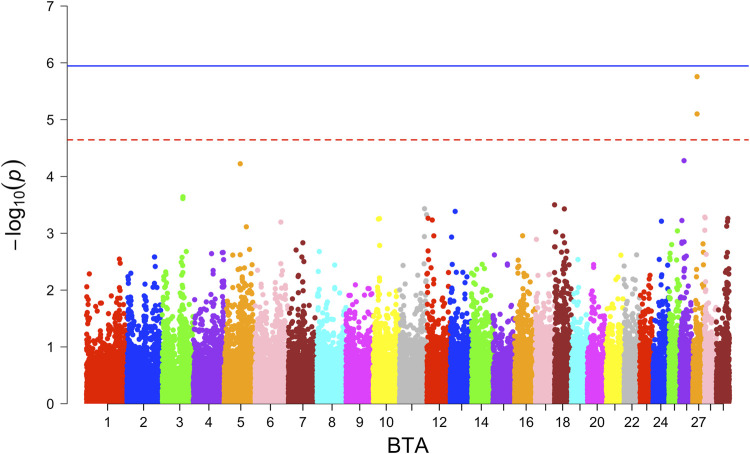
Manhattan plot for Belgian Blue crossbred population GWAS for gestation length.

For GL, two SNPs with suggestive associations were observed for WBB based on the additive model (Figure 4), while no associations were observed with either the additive or dominance model in the combined and ANG crossbreds. No haplotype block with significant association was identified across all crossbreds.

#### Parental origin of haplotype blocks

For the haplotype blocks along with the presence of the gene in the genomic region, a comparison of the block with the respective allelic pattern was made to the maternal and paternal blocks. The majority of the blocks for the BW could be traced back to the HOL breed but for blocks with suggestive association, unique blocks were identified for the parental breeds (Table 6; Supplementary Table S5).

**TABLE 6:** Genes encoded with significantly associated SNPs.

Trait	SNP	Position (bp)	Genes
BW	BTB-01601414	66,262,623	*GABRG1*
BW	Hapmap59322-rs29015787	66,307,093	*GABRG1*
BW	Hapmap27072-BTC-033816	38,133,743	*SPP1*
CD	BTB-00251468	42,057,261	KCNIP4

## Discussion

### Heritability of traits

The study used two distinctive models to estimate heritability for BW, CD and GL. The effect of crossbreeding on heritability estimates depends upon the population and varies from trait to trait, but generally slightly lower heritability is expected for crossbred populations (Wientjes and Calus, 2017). This trend is also observed in the current study where BW heritability for WBB (0.26) crossbreds is lower compared to the purebred WBB (0.38) calves (Mota et al., 2017). However, heritability for ANG crossbred is slightly higher (0.36) compared to purebred ANG (0.33) (Torres-Vázquez et al., 2018). Non-additive genetic variance is expected to be higher in crossbred populations resulting in lower estimates for heritability (Fuerst and Solkneri, 1994). In the current study heritability estimates for CD varied from 0.11 (0.03) in the combined population to 0.07 for ANG and WBB BoD calves. These estimates are closer to the one reported in British herds, where heritability for CD in BoD crossbreds is reported to be 0.09 (0.01) (McGuirk et al., 1998), but lower to the once reported for purebred ANG (0.21) and WBB (0.25–0.34) breed (Cubas et al., 1991; Vanderick et al., 2017).

Heritability for GL varied from medium 0.36 (0.04) in combined crossbreds to high in ANG BoD 0.50 (0.11). The estimates for WBB and ANG BoD are closer to the purebred counterparts with 0.33 (0.04) for WBB and 0.59 (0.01) for ANG calves (Mota et al., 2017; Gilleland et al., 2021). A strong influence of the sire breed on gestation length is expected and can be the reason for the higher heritability estimates of ANG BoD calves (Haile-Mariam and Pryce, 2019). Heritability estimates can vary across the populations based on traits under observation, but generally traits with higher *h* ^2^ in purebred animals will also have higher *h* ^2^ in crossbred population (Wientjes and Calus, 2017). For the traits with dominant genes, crossbred animals may have higher additive genetic variance compared to the purebred animals (Wei et al., 1991). Comparison across purebred and crossbred populations is challenging due to the higher environmental variance in crossbred populations due to the scale effect (Habier et al., 2007), although no general trend is observed in this regard (Wientjes and Calus, 2017). Compared to purebred animals, the less controlled environmental conditions and greater variability in farming practices associated with crossbred animals may contribute to the observed differences in this regard (Wei and Van der Werf, 1995).

### GWAS based on additive, dominance and haplotypes

Utilization of GWAS can help to understand the genetic architecture of relevant traits. In this study, GWAS was performed using three different models for BW, CD and GL in BoD crossbreds. GWAS based on haplotypes outperformed the SNP-based GWAS in terms of finding the associated genes for BW, but no significant differences were observed for GL and CD. The addition of dominance effects in the GWAS model was beneficial for BW only in terms of finding genomic regions with significant or suggestive associations. Based on SNP and haplotype GWAS, in total five genes with significant association and seven genes with suggestive association were identified for the BW trait. Role of these genes varied from ion-channels regulation to regulation of mineralization process of the bone. *GABRG1* gene, identified through significant SNP association is responsible for the regulation of the ion channel and receptor activity along with the influence on puberty development (Tahir et al., 2021). The association of this gene with milk yield has also been previously reported in the Holstein population (Pedrosa et al., 2021). *SPP1* gene identified based on the dominance model encodes for multifunctional cytokines (Matsumoto et al., 2019) and is vital for bone mineralization process (Rodriguez et al., 2014). Association between *SPP1* gene and growth traits including yearling weight, live weight and average daily gain has been suggested in American beef crossbred herds (White et al., 2007). Moreover, the influence of this gene on the birth weight (Allan et al., 2007) and carcass weight has been established in beef cattle (Matsumoto et al., 2019). For the genes identified through haplotypes-based GWAS, the influence of *CCSER1*, *KCNIP4*, and *LCORL* genes has been reported on BW and growth traits in the Angus breed (Xia et al., 2017; Smith et al., 2022). The *CCSER1* gene, controlling mitosis has association with milk yield in Holstein cows (Teng et al., 2023). Similarly, *PPARGC1A* gene, influencing fat deposition and energy metabolism has been associated with BW (Pasandideh, 2020). Previously *FAM13A* gene with regulatory role in metabolism has already been described in the Holstein breed for association with bone structure (Niu et al., 2021; Dominguez-Castaño et al., 2024) Moreover, associations between *PPARGC1A*, a gene with significant role in fat metabolism (Komisarek and Walendowska, 2012) and yearling weight along with carcass traits have been observed in beef cattle (Fonseca et al., 2015). In this study, higher statistical power was observed for Haplotype-based GWAS in identifying associations and candidate regions. Similar results have been previously described in various species, including cattle (Pryce et al., 2010; Barendse and Schneider, 2011) and pigs (Sato et al., 2016). To some extent these differences can be attributed to the window size for haplotypes and the number of markers per haplotype can potentially influence the accuracy of QTL mapping (Calus et al., 2009). In contrast to aforementioned studies, SNP-based GWAS was found to be more efficient for carcass traits in Simmental cattle (Wu et al., 2014).

### Parental origin of haplotype blocks

Interest in the approaches in the partitioning of crossbred genome has gained significant interest in the recent years for the crossbred evaluation. Various methods with varying degree of success have been developed including breed base representation (BBR) and breed of origin of the allele (BOA) (VanRaden et al., 2020; Eiríksson et al., 2021b; Guillenea et al., 2023). Despite the advancements in the statistical models and the increased number of genotyped animals, 100% precise estimation of the share of the genome of each parent in the crossbred population is difficult due to the shared DNA content between breeds (VanRaden et al., 2020). In this study, despite the partial success of assigning haplotypes to the parental breeds, complete segregation of the haplotypes of the crossbred calves was not successful. For most of the haplotypes with significant associations, the origin of the haplotypes was traced back to both parental haplotypes, though six breed-specific haplotypes with similar pattern of alleles were identified for ANG (n = 3) and WBB (n = 3) breeds. All of these six haplotypes were also present in HOL population (Supplementary Table S5).

### Limitations of the study

A key limitation of the current study was the insufficient number of genotyped dams as this may reduce the accuracy of haplotype phasing. Moreover, for calving difficulty and gestation length, maternal influence is significant, and unavailability of sufficient number of maternal genotypes and phenotype records can be a limiting factor in this regard. Furthermore, the unequal number of crossbreds from two beef breeds can have impact on the accuracy of genetic parameters and association studies.

## Conclusion

Models based on combined populations resulted in the identification of more associated genes compared to the separate crossbred populations. Heritability estimates for BW and GL in BoD crossbred populations were similar to those found in purebred populations. However, the heritability estimates for CD were comparatively lower. Furthermore, combining crossbred populations with at least one common parent breed can be a viable strategy for estimation of SNP based genetic parameters and association studies. Incorporating dominance effects in both GWAS and heritability estimates resulted in modest improvements in model performance. Additionally, haplotype-based GWAS proved better in identifying genes associated with traits in crossbred animals compared to traditional SNP-based approaches. The origin of the majority of haplotype blocks with significant and suggestive associations can be traced back to the HOL breed, though some sire’s specific blocks were also identified in the current study.

## Data Availability

The original contributions presented in the study are publicly available. This data can be found here: Ahmed (2025).
