# Supplementary material for: Genomic-based genetic parameters and genome-wide association studies for productive and reproductive traits in Beef-on-Dairy crossbreds
Source: Front Genet. 2025 Jun 5;16:1530310. doi: 10.3389/fgene.2025.1530310 (PMC12177717; doi:10.3389/fgene.2025.1530310)
Supplement: Supplementary file 1 [file DataSheet1.docx]

Supplementary Material

**Table S1** SNPs removed during quality control per breed.

| Breed | MAF | HWE | Missing | SNPs (GWAS) |
| --- | --- | --- | --- | --- |
| Combined | 655 | 121 | 0 | 37468 |
| ANG | 0 | 0 | 0 | 38244 |
| WBB | 0 | 0 | 0 | 38244 |

**Table S2** Distribution of sire breed and dams

| Breed | Nr calves  (genotyped) | Nr sires  (genotyped) | Nr dams  (genotyped) |
| --- | --- | --- | --- |
| ANG | 852 (801) | 11 (7) | 837 (286) |
| WBB | 2981 (2729) | 35 (15) | 2802 (749) |

**Table S3** Genetic parameters for birth weight, calving ease and gestation length

| Breed | Trait |  | MODEL | | | | | |
| --- | --- | --- | --- | --- | --- | --- | --- | --- |
|  |  | Additive | | |  | Dominance | | |
|  |  | V_a_ (S.E) | | V_e_ (S.E) | V_a_ (S.E) | | V_d_ (S.E) | V_e_ (S.E) |
| **Combined** | BW | 11.56 (1.59) | | 28.50 (1.18) | 10.56 (1.56) | | 0 | 29.27 (2.19) |
|  | CE | 0.01 (0.002) | | 0.08 (0.002) | 0.01 (0.003) | | 0.01 (0.01) | 0.07 (0.01) |
|  | GL | 7.39 (0.87) | | 13.02 (0.59) | 7.39 (0.89) | | 0.02 (1.15) | 13.00 (1.06) |
| **ANG** | BW | 12.43 (4.50) | | 21.82 (3.30) | 11.87 (4.73) | | 4.30 (8.2) | 18.96 (6.15) |
|  | CE | 0.02 (0.01) | | 0.07 (0.01) | 0.02 (0.01) | | 0.03 (0.02) | 0.06 (0.02) |
|  | GL | 9.86 (2.64) | | 9.72 (1.78) | 8.49 (2.69) | | 0 | 10.12 (2.91) |
| **WBB** | BW | 10.87 (1.92) | | 30.15 (1.50) | 10.54 (1.97) | | 0 | 30.36 (2.54) |
|  | CE | 0.01 (0.003) | | 0.08 (0.003) | 0.01 (0.003) | | 0.01 (0.01) | 0.07 (0.01) |
|  | GL | 7.26 (1.04) | | 13.37 (0.73) | 7.21 (1.08) | | 0 | 13.19 (1.26) |

**Table S4** Suggestive association of haplotypes with birth weight and calving difficulty

| Breed | Trait | BTA | Position | Gene |
| --- | --- | --- | --- | --- |
| **COM** | CD | 6 | 55496362 - 55594668 | - |
| **COM, WBB** | BW | 6 | 38063313 - 38203273 | SPP1 |
| **COM** | BW | 6 | 66209350 - 66307093 | GABRG1 |
| **ANG** | BW | 6 | 37704254 - 37868743 | HERC6 |
| **ANG** | BW | 6 | 36770471 - 36930214 | - |
| **ANG** | BW | 6 | 36930214 - 37104193 | LOC104972722 |
| **COM, WBB** | BW | 6 | 79126321 - 79379568 | ADGRL3 |
| **ANG** | BW | 6 | 44850990 - 44991839 | PPARGC1A |
| **COM** | BW | 6 | 48216479 - 48423262 | - |
| **ANG** | BW | 6 | 36277967 - 36580568 | SNCA |

**Table S5** Parental origin of haplotypes with suggestive association with traits.

| Breed | Block Position (bp) | Origin |
| --- | --- | --- |
| **COM** | 55496362 - 55594668 | - |
| **COM, WBB** | 38063313 - 38203273 | WBB, HOL |
| **COM** | 66209350 - 66307093 | ANG, WBB, HOL |
| **ANG** | 37704254 - 37868743 | ANG, HOL |
| **ANG** | 36770471 - 36930214 | ANG, WBB, HOL |
| **ANG** | 36930214 - 37104193 | ANG, WBB, HOL |
| **COM, WBB** | 79126321 - 79379568 | WBB, HOL |
| **ANG** | 44850990 - 44991839 | ANG, HOL |
| **COM** | 48216479 - 48423262 | ANG, HOL |
| **ANG** | 36277967 - 36580568 | WBB, HOL |


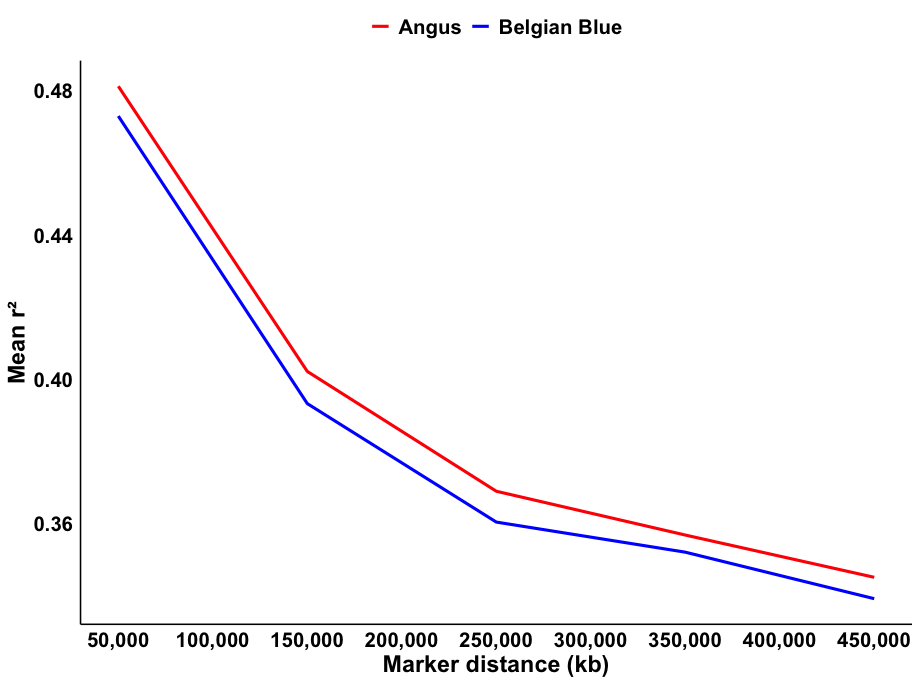


**Figure S1** LD decay of crossbred populations.

**Figure S2** Distribution of parity number of the dam for ANG and WBB crossbreds.


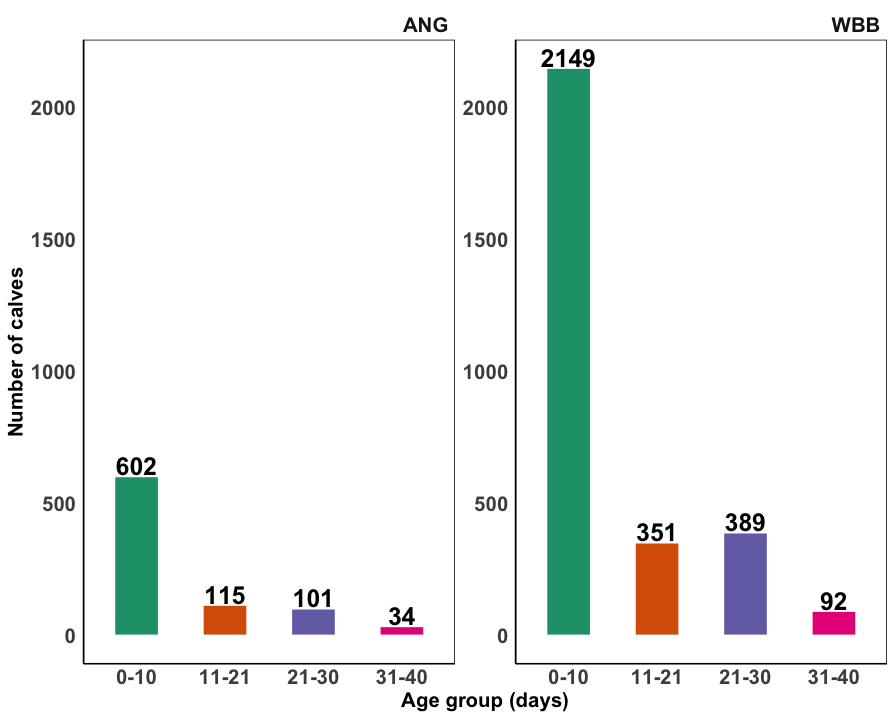


**Figure S3** Age distribution of crossbred population.


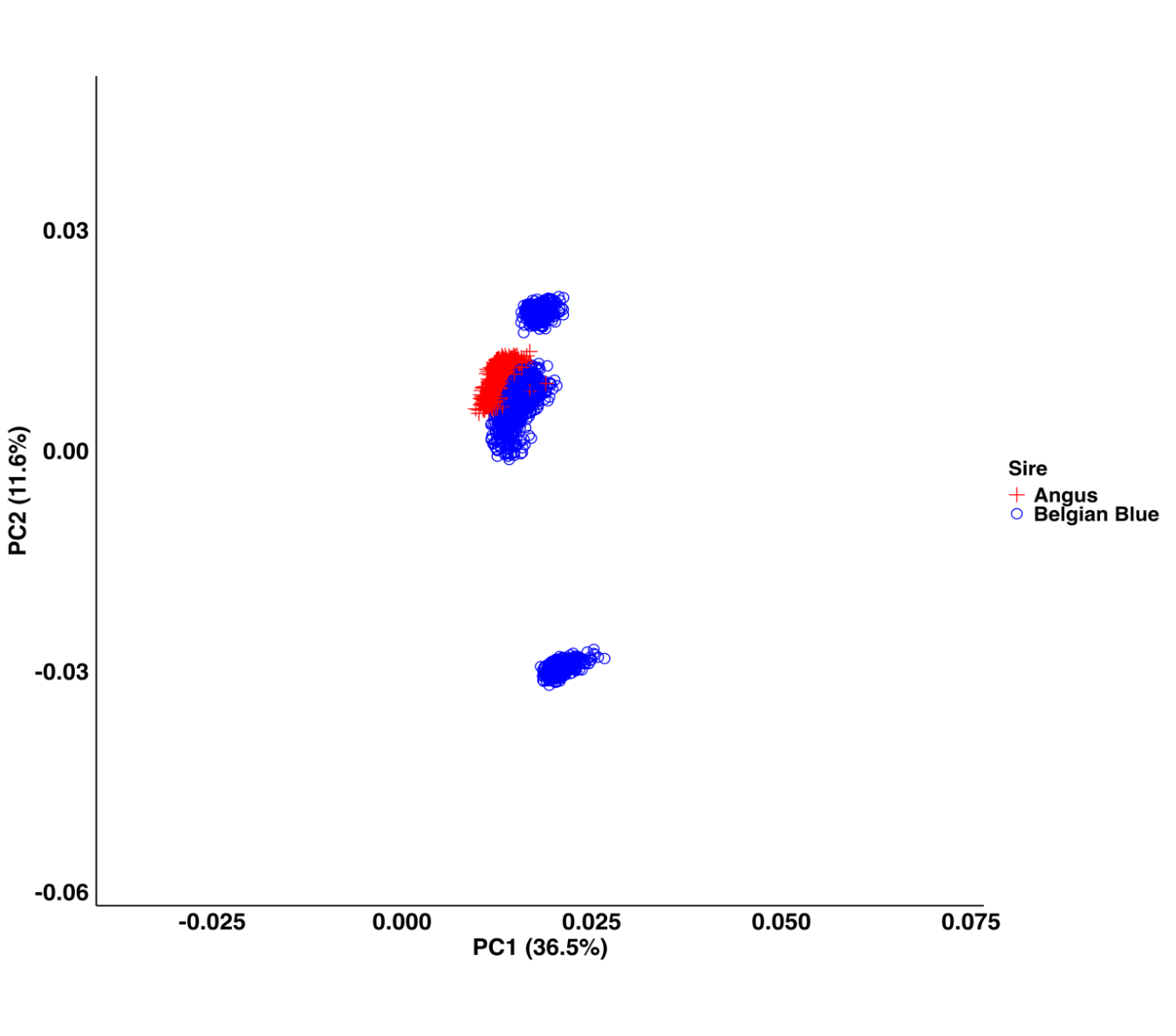


**Figure S4** PC1 and PC2 of population structure of crossbred calves


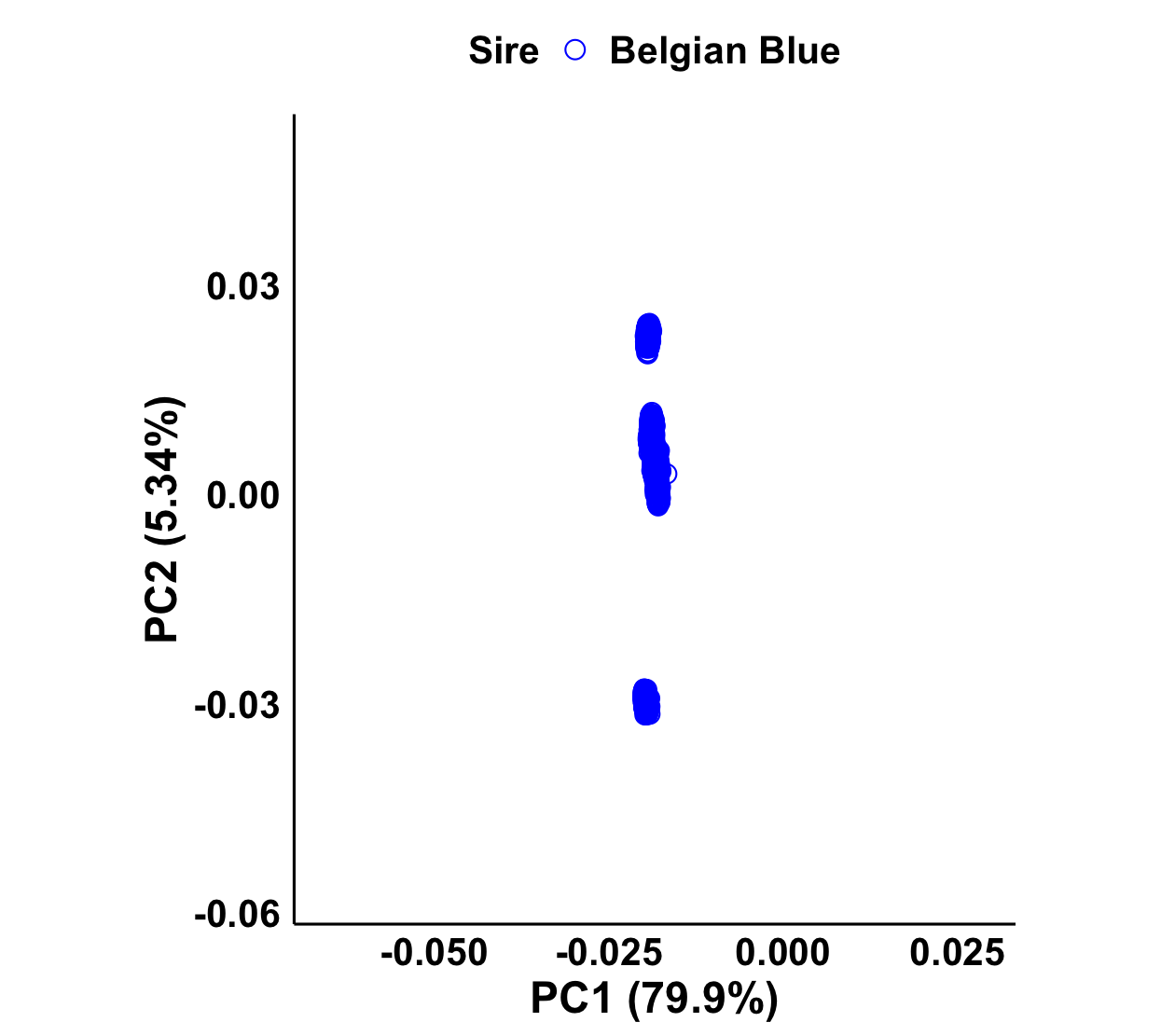


**Figure S5** PC1 vs PC2 of population structure of Belgian blue crossbreds.


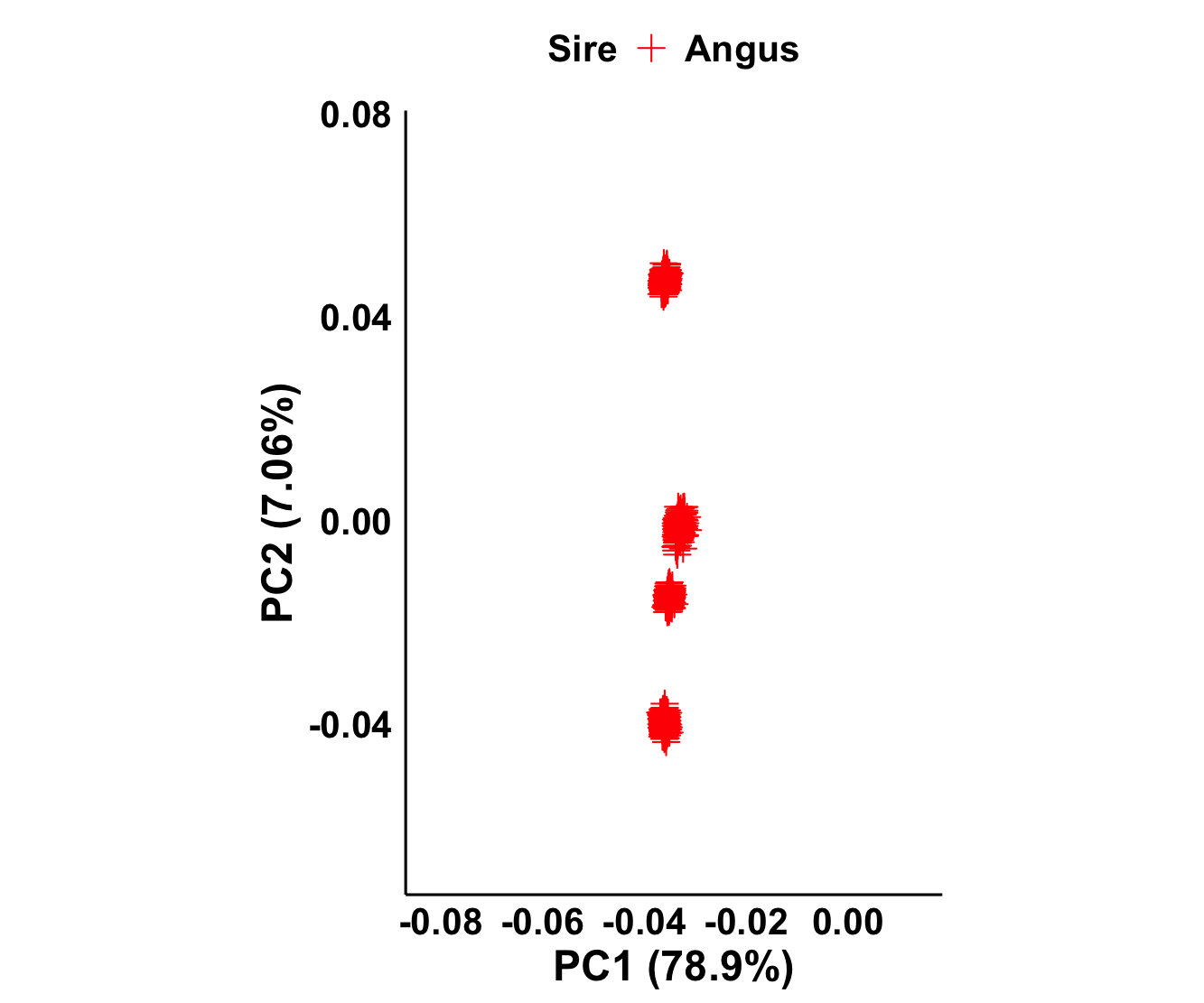


**Figure S6** PC1 vs PC2 of population structure of Angus crossbreds.

**
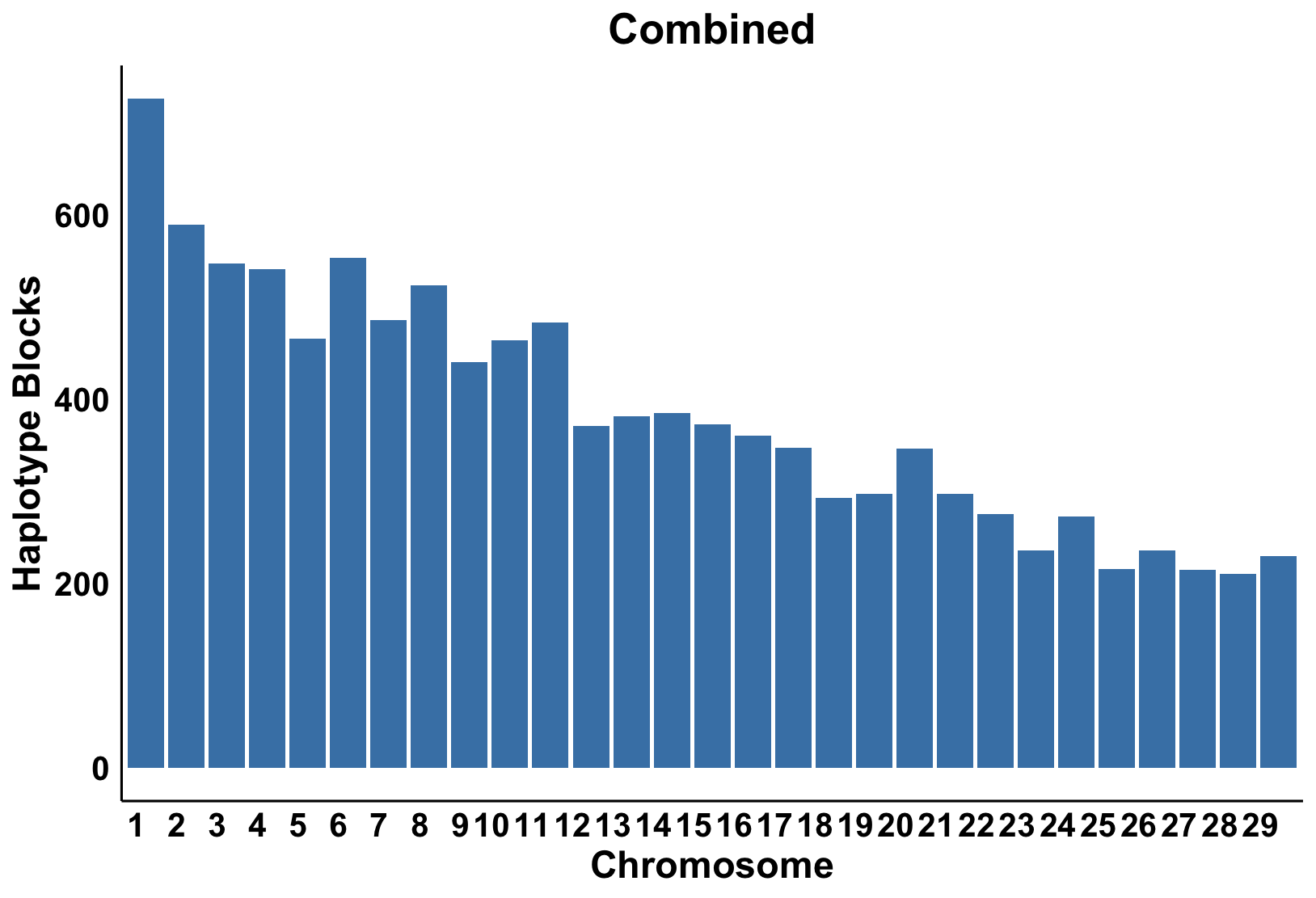
**

**Figure S7** Haplotype blocks distribution per chromosome.

**Figure S8** Haplotype based GWAS for birth weight in a) Angus 2) Belgian Blue and 3) Combined population

**
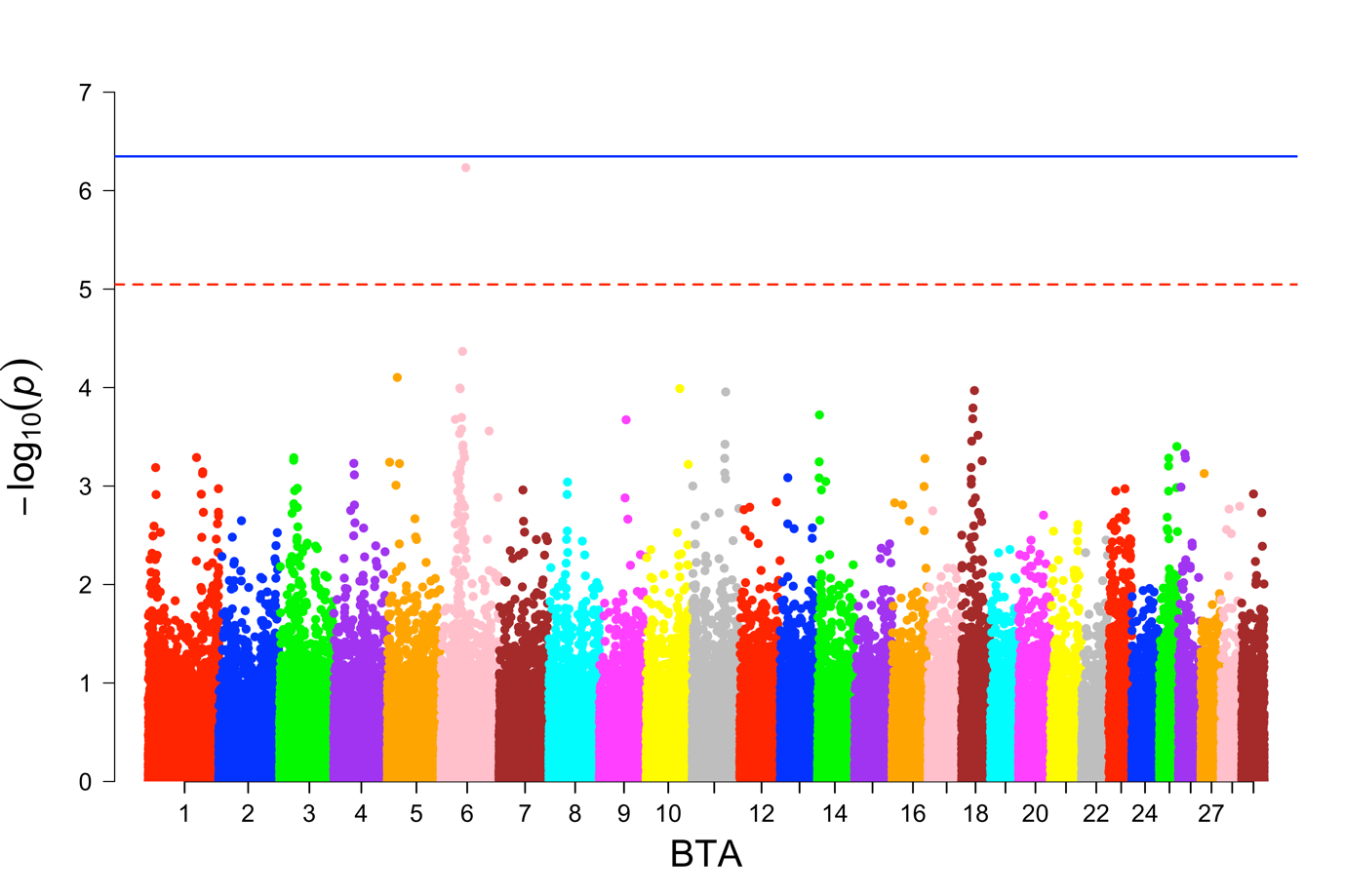
**

**Figure S9** Haplotype based GWAS for calving difficulty in Combined population
